# Airborne signals synchronize the defenses of neighboring plants in response to touch

**DOI:** 10.1093/jxb/ery375

**Published:** 2018-10-31

**Authors:** Dimitrije Markovic, Ilaria Colzi, Cosimo Taiti, Swayamjit Ray, Romain Scalone, Jared Gregory Ali, Stefano Mancuso, Velemir Ninkovic

**Affiliations:** 1 Department of Crop Production Ecology, Swedish University of Agricultural Sciences, Uppsala, Sweden; 2 Faculty of Agriculture, University of Banja Luka, Banja Luka, Bosnia and Herzegovina; 3 Department of Biology, University of Florence, Florence, Italy; 4 Department of Entomology, The Pennsylvania State University, University Park, PA, USA; 5 Department of Ecology, Swedish University of Agricultural Sciences, Uppsala, Sweden

**Keywords:** Gene expression, host plant acceptance, plant–plant communication, priming, *Rhopalosiphum padi*, terpenes, touch, volatile organic compounds, *Zea mays*

## Abstract

Plants activate defense-related pathways in response to subtle abiotic or biotic disturbances, changing their volatile profile rapidly. How such perturbations reach and potentially affect neighboring plants is less understood. We evaluated whether brief and light touching had a cascade effect on the profile of volatiles and gene expression of the focal plant and a neighboring untouched plant. Within minutes after contact, *Zea mays* showed an up-regulation of certain defense genes and increased the emission of specific volatiles that primed neighboring plants, making them less attractive for aphids. Exposure to volatiles from touched plants activated many of the same defense-related genes in non-touched neighboring plants, demonstrating a transcriptional mirroring effect for expression of genes up-regulated by brief contact. Perception of so-far-overlooked touch-induced volatile organic compounds was of ecological significance as these volatiles are directly involved in plant–plant communication as an effective trigger for rapid defense synchronization among nearby plants. Our findings shed new light on mechanisms of plant responses to mechanical contact at the molecular level and on the ecological role of induced volatiles as airborne signals in plant–plant interactions.

## Teaser

Gentle touch is an effective stimulus in the activation of transcription of several defense-related genes involved in the emission of specific volatiles. The volatiles activate the same genes in neighboring plants.

## Introduction

The sessile nature of plants has led to a dependence on their ability to sense, process, and respond to a range of different types of environmental stimuli. Contact initiated by conditions such as wind, rain, or nearby organisms is one of the most common stimuli experienced by plants (Moran & Cipollini, 1999; Anten *et al.*, 2010). Plants have evolved very sensitive mechanisms that allow them to perceive touch and launch appropriate responses that include alterations in gene expression (Jaffe *et al.*, 2002; Telewski, 2006; Chehab *et al.*, 2012). It has been shown that alteration in gene expression in plants like Arabidopsis can also rapidly occur within minutes after touch (Lee *et al.*, 2005). Some of the genes up-regulated after touch includes those that have been implicated in disease resistance and defense responses against insect herbivores (Lee *et al.*, 2005; Chehab *et al.*, 2012). Activation of defense-related genes is followed by the emission of a large variety of signaling substances not present in the profiles of untouched plants (Braam, 2005). Recent studies have shown changes in volatiles released by touched plants (Chehab *et al.*, 2012; Markovic *et al.*, 2014, 2016), but the precise timing of volatile emission upon the onset of mechanical stress is still unknown.

The ecological context of plant defense responses in receivers after exposure to volatiles emitted by infested or damaged plants has been examined and shown to have clear adaptive significance (Dicke & Bruin, 2001; Engelberth *et al.*, 2004; Kessler *et al.*, 2006; Heil & Karban, 2010; Ramadan *et al.*, 2011). Recently, it has become apparent that chemical interactions between undamaged plants may also carry important information that can affect trophic interactions (Ninkovic *et al.*, 2013). Plants’ ability to eavesdrop on the status of their neighbors may facilitate adaptive responses (Ninkovic *et al.*, 2016). However, it is unknown how volatiles released from a plant that has simply been touched can influence responses in its conspecific neighbors. Furthermore, would the response of a receiving plant mimic the defensive response of the emitting plant? A plant that comes into contact with a non-self-stimulus might be predicted to have a response to this foreign body, as it represents a potential threat or future competitor. Therefore, would a plant that is receiving contact stimuli elicit a response that could affect a neighbor, and would this information carry with it the severity of this threat causing a corresponding response in the neighbor? To date, potential ecological consequences of touch-induced volatiles in direct chemical interaction between plants as mechanisms contributing to suppression of pests have not been elucidated. Due to their particularly close interaction with plants, aphids are an excellent herbivore model for detecting changes in plant status following chemical interactions among plants (Pettersson *et al.*, 2017). Aphids make considerable use of chemical information in host plant selection or avoidance (Birkett *et al.*, 2000; Eigenbrode *et al.*, 2002; Pettersson *et al.*, 2017).

Our study was conducted to gain further insight into the temporal response of plants to touch and the associated ecological implications. For this purpose, we evaluated (i) changes in gene expression and volatile emission, (ii) whether volatiles released by touched plants carrying long-range information prime activation of the same stress-related genes in neighboring plants, and (iii) whether induced responses in nearby plants may be detected by herbivorous insects.

## Material and methods

### Plant material

As a model plant, we used maize *Zea mays* L. cultivar Delprim obtained from Delley Seeds and Plants Ltd (Switzerland). Prior to sowing, seeds were sterilized in 70% ethanol for 3 min and rinsed two times in deionized water; then seeds spent 15 min in a 1:1 solution of chlorine and water, and were rinsed again four times in deionized water. Plants were grown in plastic pots (9 × 9×7 cm) in potting soil (Hasselfors Garden, Sweden) with one seed per pot in a glasshouse at 18–22 °C, with a 16 h light–8 h dark cycle, light intensity of 150 µmol m^−2^ s^−1^, and relative humidity of 60 ± 10%; they were watered via an automated water drip system delivering 22 ml daily at 08.00 h (2 h into the photoperiod). Natural light was supplemented by light from HQIE lamps. Six days after sowing at the early two-leaf stage, all plants were selected for uniformity in size and moved into clear Perspex cages.

### Insects

Bird cherry-oat aphids, *Rhopalosiphum padi* L., were reared on barley, *Hordeum vulgare* L. cultivar Golf, in multi-clonal cultures in a glasshouse with the same conditions as for the plants. Aphids used in the experiments were wingless, mixed-instar individuals, collected from the cultures 30 min prior to bioassay.

### Touching treatments

Plants were grown in clear Perspex cages divided into two separate chambers (each 10 × 10 × 40 cm), and connected by an opening (7 cm in diameter) in the dividing wall (Ninkovic *et al.*, 2002), which prevented plants from interacting with each other in any way. Air entered the front chamber through an opening in the cage wall (7 cm in diameter) and was extracted from the rear chamber through a tube attached to a vacuum tank and vented outside the room by an electric fan. Airflow through the cages was 1.3 liters min^−1^. On each test occasion, emitting plants were placed in the front chambers while receiving plants were placed in consecutive rear receiving chambers. Thus, the front chamber in each block contained either one touched or one untouched plant adjacent to a plant in a receiving chamber (see Supplementary Fig. S1 at *JXB* online). Cages within blocks were distributed spatially to compensate any small but potentially important variations in abiotic conditions. Each of the treatments was repeated 18 times and they were randomly distributed in different blocks.

To identify activation of stress-related genes that could further affect emission of volatiles, we applied contact treatments with a soft brush with 3.5 cm-long fibers and a surface area of 7 cm^2^ at the point of contact. After 24 h of acclimation in the chambers, a maize leaf was carefully touched from the leaf base to the top, using previously described methods (Montgomery *et al.*, 2003; Liu *et al.*, 2007; Anten *et al.*, 2010; Markovic *et al.*, 2016). This treatment with a brush has been used to simulate the mechanical interactions between plants (Elhakeem *et al.*, 2018). Treated plants were touched in the morning for 1 min per day over a period of 6 d. Experiments were limited to 6 d of growth to avoid plants becoming too large and contacting the top of the chambers.

### Volatile organic compound analysis by proton-transfer-reaction time-of-flight mass spectrometry

Volatile organic compound (VOC) measurements were performed using a commercial proton-transfer-reaction time-of-flight (PTR-TOF) mass spectrometer (PTR-TOF 8000 model; Ionicon Analytik GmbH, Innsbruck, Austria) and were carried out both on young maize plants at the two-leaf stage, still untouched, and on plants previously subjected to touching treatment for 6 d. The PTR-TOF 8000 has been described in detail in several publications (Jordan *et al.*, 2009), and therefore, in this paper, only a brief description will be provided. This tool is an analytical instrument with high sensitivity (>200 cps/ppbv) and high mass resolution (<8000 *m*/Δ*m*), providing a chemical formula identification for each compound (Lanza *et al.*, 2015).

VOC measurements were performed using a system similar to that previously described (Taiti *et al.*, 2016) with some modifications. Briefly, each plant was covered with a glass jar equipped with a small opening to allow contact treatments and two Teflon tubes located on the air inlet and outlet, respectively. The shoot headspace of touched and untouched plants was sampled directly from the glass jar through the outlet tube connected to the PTR-TOF-MS. The inlet tube was connected to a zero-air generator (Peak Scientific) to maintain a constant flow of hydrocarbon-free air into the jar during the headspace collection. Care was taken to maintain constant temperature and humidity during measurements as chemical reactions are very sensitive to such parameter changes (Mancuso *et al.*, 2015).

VOC analysis of each plant was performed for a minimum of 10 min under control conditions, after which the contact treatment was applied for 1 min as described above. Each measurement lasted, in total, 150 min. VOC emission of control plants (untouched) was also recorded along the same time period. Moreover, VOCs in the headspace of an empty glass jar were analysed as a blank control. The time of sampling for each TOF acquisition channel was 0.1 ns, for a mass spectrum between *m*/*z* 30 and 210. The instrument was set as follows: inlet flux to 50 sscm, drift pressure of 2.20 mbar, temperature of 50 °C, voltage of 600 V, extraction voltage at the end of tube (*U*_dx_) of 35 V, which corresponds to an *E*/*N* value of 140 Td, which provides the necessary balance between excessive water cluster formation and ion fragmentation products (Pang, 2015).

Internal calibration was performed off-line after dead time correction based on *m*/*z*=29.997 (NO), *m*/*z*=59.049 (C_2_H_5_O_2_), *m*/*z*=180.937 (C_6_H_4_C_l3_) to obtain high mass accuracy. Raw data were acquired with TofDaq software (Tofwerk AG, Switzerland), peak quantification data were corrected with the duty cycle, and the signals were normalized to the primary ion signal (count per second (cps) to normalized count per second (ncps)) (Taiti *et al.*, 2015). For the identified signals, all of the *m*/*z* were tentatively assigned to the mass formulae reported, relying on high instrumental mass accuracy and resolution. Moreover, the tentative identifications through the integration of previous knowledge of the VOCs emitted by plants, where analyses were performed with PTR-MS instruments, improved (Maleknia *et al.*, 2007; Kim *et al.*, 2010).

### Sample collection

Leaf tissue was collected from plants at either 6 min or 100 min after the first and last contact treatments. Maize leaves were then sampled without touching the plants, but by moving the pots to a BIOREBA^TM^ extraction bag and allowing the cut sample to fall into the bag before being placed in liquid nitrogen. Approximately 3 cm of leaf tissue was removed using sterile scissors. Tissue was collected from five plants from each treatment: touched plant, T; control untouched plant, C; neighbors exposed to volatiles from touched plant, ET; and neighbors exposed to volatiles from control untouched plants, EC (see Supplementary Fig. S1).

### RNA preparation and quantitative real-time PCR

Each frozen sample bag with an individual leaf inside was stored at –70 °C before RNA extraction. RNA was extracted from 100 mg of leaf tissue after being ground in liquid nitrogen using Trizol reagent (Life Technologies, USA). The manufacturer’s protocol was modified and 8 M lithium chloride was used to remove genomic DNA contamination from the extracted RNA. Ten micrograms of the total RNA from each sample was then freeze-dried in GenTegra tubes (GenTegra, USA) for transport. RNA was dissolved in nuclease-free water, quantified and 1 µg of RNA was used from each sample to generate cDNA using a High Capacity Reverse Transcriptase kit (Life Technologies, USA). Primers for real-time PCR were generated using Primer 3 express software and validated with BLAST searches in NCBI and Maize Genome databases. The lists of primers that were used are provided in Supplementary Table S1. Quantitative real-time PCR (qRT-PCR) was performed using SYBR green reagent in the 7900 HT Fast Real-time PCR System (Applied Biosciences, USA). Actin was used as endogenous control for each sample and relative quantification (RQ) values were calculated using the ΔΔ*C*_T_ method. The average gene expression of five plants, from which plant tissue were collected before the start of experiment, were used as a calibrator for the RQ calculations for gene expression.

Three categories of genes were investigated: (i) genes regulating the early defense of the plant (e.g. the superoxide dismutase gene, *SOD*), (ii) genes involved in the production of chemical defense against herbivores (e.g. the wound-induced protease inhibitor 1 gene, *WIP1*), and (iii) genes involved in the production of volatile compounds priming neighboring plants or attracting natural enemies of herbivores (e.g. the terpene synthase 2 gene, *TPS2*). The names and the function of the genes as well as the primers used for their amplification by qRT-PCR are listed in Table S1.

### Aphid plant acceptance

A no-choice settling test was used to measure aphid acceptance of maize plants using previously described methods, summarized below (Ninkovic *et al.*, 2002). All maize plants were tested 24 h after the end of the sixth contact treatment. The second maize leaf was placed inside a transparent 100 ml polystyrene tube (diameter 2.5 cm, length 25 cm). The upper end of the tube was sealed with a nylon net and the lower end was plugged with a plastic sponge through which the leaf entered via a slit. Ten wingless *R. padi* (larval instars 2–4) were placed inside a polystyrene tube around the maize leaf, and the number of aphids settled (not walking) on the leaf was recorded after 2 h, which is sufficient time for aphids to settle and reach the phloem (Prado & Tjallingii, 1997). The results were expressed as the proportion of aphids originally introduced to ensure that aphids that were able to escape from the tubes did not bias the results. The proportions of settling aphids were analysed with generalized linear mixed models (GLMs) with binomial error distribution in lme4 (Bates *et al.*, 2015) in R (R Core Team, 2015). The Tukey test was used for *post hoc* comparisons (SAS v. 9.4; SAS Institute, Cary, NC, USA). There were 18 replicates of T, C, ET, and EC plants.

## Results

### Volatile emission

**Fig. 1.**
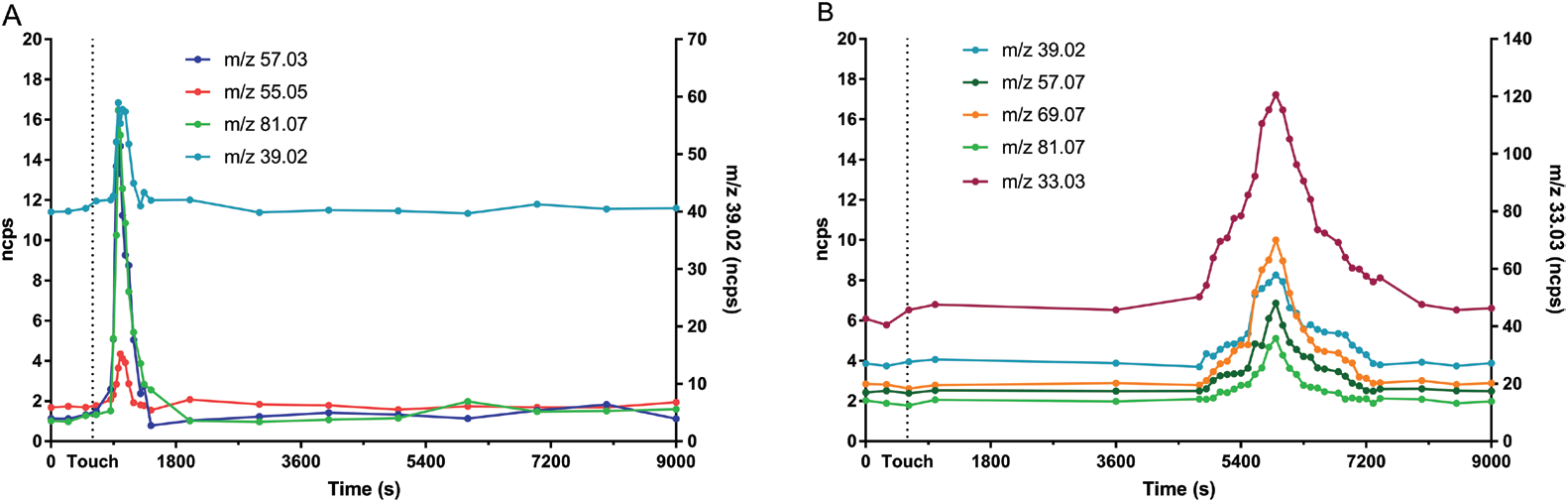
Real-time VOC emission of young maize plants at the two-leaf stage (A) touched for the first time and (B) touched for 6 d. Touching treatment is indicated by a vertical dotted line (time 600 s).

PTR-TOF-MS analysis showed real-time VOC emission from maize plants before and after the contact treatments. The time course of VOCs emitted by young maize plants at the two-leaf stage touched for the first time demonstrated a transient increase of emission of the volatile compounds after contact for compound of *m*/*z* 39.02 (tentative identification (TI): isoprene fragment), *m*/*z* 55.05 (TI: C4 aldehydes fragment), *m*/*z* 57.03 (TI: (*E*)-3-hexenal), and *m*/*z* 81.07 (TI: terpene fragment) (Fig. 1A). The initial burst of these compounds exceeded the constitutive emission and started about 240 s after the mechanical stimulus was applied and lasted about 300 s, reaching the maximum peak after 390–400 s (Fig. 1A).

Plants touched for 1 min d^−1^ for 6 d prior to PTR-TOF-MS analysis (Fig. 1B) seemed to trigger a slower but longer increase of some protonated masses, *m*/*z* 33.03 (TI: methanol), *m*/*z* 39.02 (TI: isoprene fragment), *m*/*z* 69.07 (TI: isoprene), *m*/*z* 57.07 (TI: alkyl fragment), and *m*/*z* 81.07 (TI: terpene fragment). The release of these masses started to increase ~4600 s after the touching treatment, peaking at 5600 s. All the detected masses lasted nearly 2500 s before reaching their constitutive emission levels.

### Gene expression

Early plant defenses are regulated by the generation of reactive oxygen species (ROS), which not only can alter the redox state of the plant and inhibit insect performance, but also may function as secondary messengers activating downstream plant defense responses (Apel & Hirt, 2004). We measured gene expression in response to touch for genes that regulate the redox state of the plant such as ascorbate peroxidase (*APX*), calcium-dependent protein kinase (*CPK11*), mitogen-activated protein kinase 1 (*MKK1*), and superoxide dismutase (*SOD*). Plants have been shown to induce *CPK11* expression in response to wounding and touch in maize (Szczegielniak *et al.*, 2012). Expression of genes for mitogen-activated kinases such as *MKK1* has also been shown to be triggered by ROS and antioxidants in maize and to affect the plants’ innate immunity (Zhang *et al.*, 2006; Gao *et al.*, 2008). Maize plants trigger expression of early defense genes such as superoxide distmutase (*SOD*) and *APX* in response to aphid attack (Sytykiewicz, 2014, 2016). In addition, we also measured transcript abundance of defense genes whose products are known to affect insect performance directly, such as proteinase inhibitors and benzoxazinoids. Expression of maize protease inhibitor (*MPI*), wound-induced protease inhibitor 1 (*WIP1*) and benzoxazineless 1 (*BX1*) in maize has also been shown to hinder aphid and caterpillar growth on plants (Ahmad *et al.*, 2011; Chuang *et al.*, 2014; Louis *et al.*, 2015). A plants can also prime its neighbors and recruit natural enemies of its insect pests through the production of VOCs such as terpenoids, green leafy volatiles, and indole through the gene products of terpene synthase 2 (*TPS2*), lipoxygenase 3 (*LOX3*), and indole glycerol phosphate lyase (*IGL1*), respectively. Therefore, we measured the transcript abundance of *TPS2*, *LOX3*, and *IGL1* in response to touch. We found that gene expression among T or ET and C or EC plants did not show much change after the first 1 min of contact. However, T plants exposed to continuous touching of 1 min d^−1^ for 6 d and neighbors exposed to their volatiles (ET plants) had significantly higher gene expression compared with C or EC plants, respectively.

**Fig. 2.**
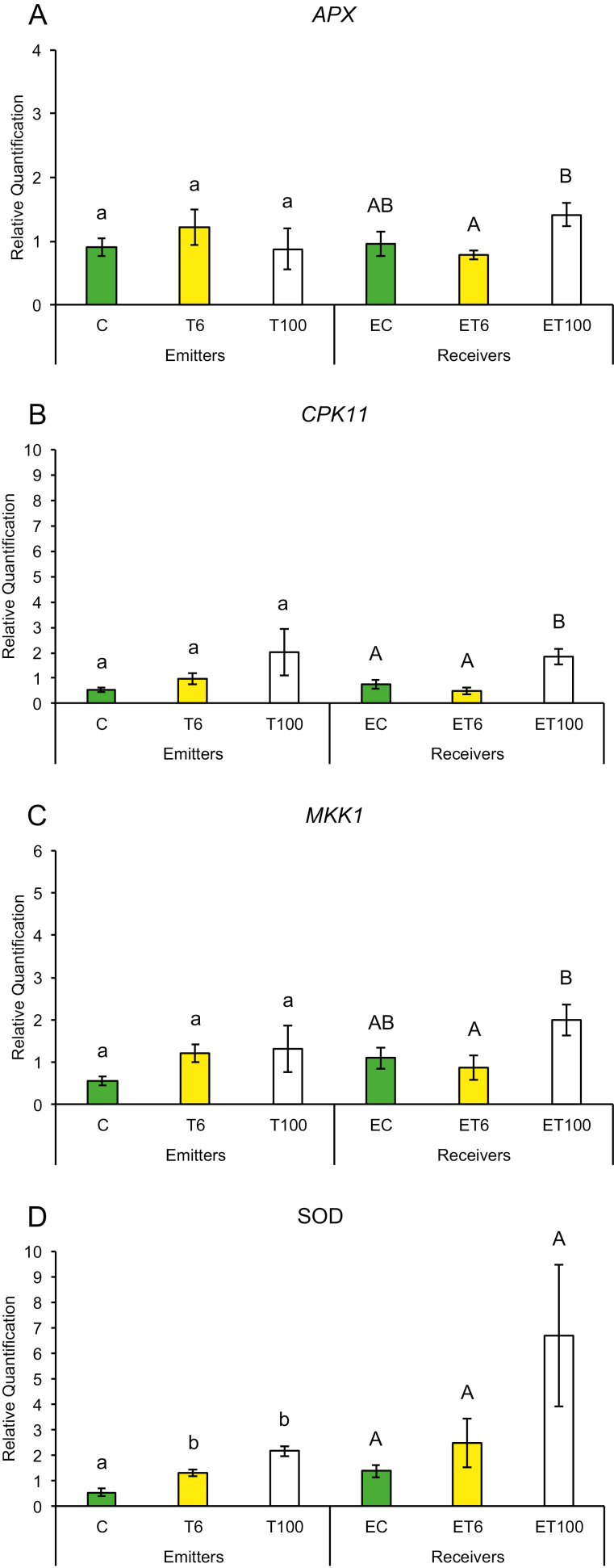
Expression profile of early plant defense-related genes after first treatment in untouched control plants (C), touched plants after 6 min (T6) and 100 min (T100), and neighbors exposed to volatiles released from untouched control plants (EC), touched plants after 6 min (ET6) and touched plants after 100 min (ET100). Different letters above each variable represent significant differences between treatments (Tukey’s test).

**Fig. 3.**
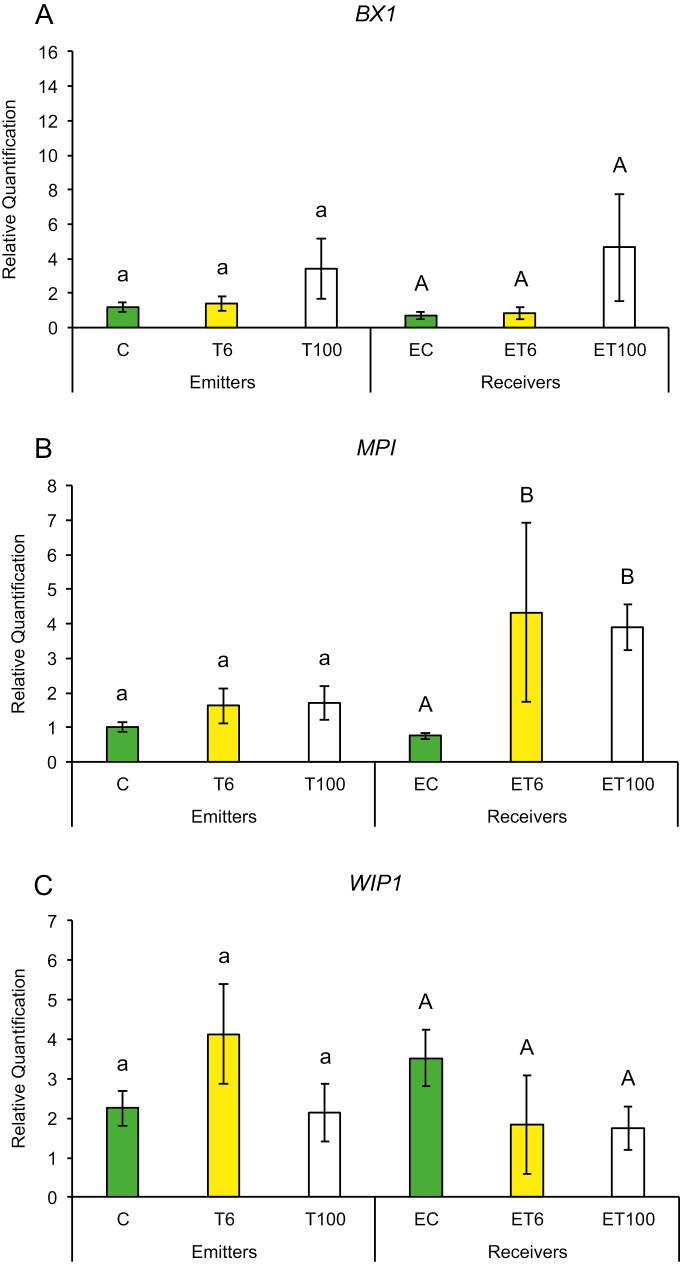
Changes in expression of genes involved in the production of chemical defense against herbivores after first treatment in untouched control plants (C), touched plants after 6 min (T6) and 100 min (T100), and neighbors exposed to volatiles released from untouched control plants (EC), touched plants after 6 min (ET6) and touched plants after 100 min (ET100). Different letters above each variable represent significant differences between treatments (Tukey’s test).

**Fig. 4.**
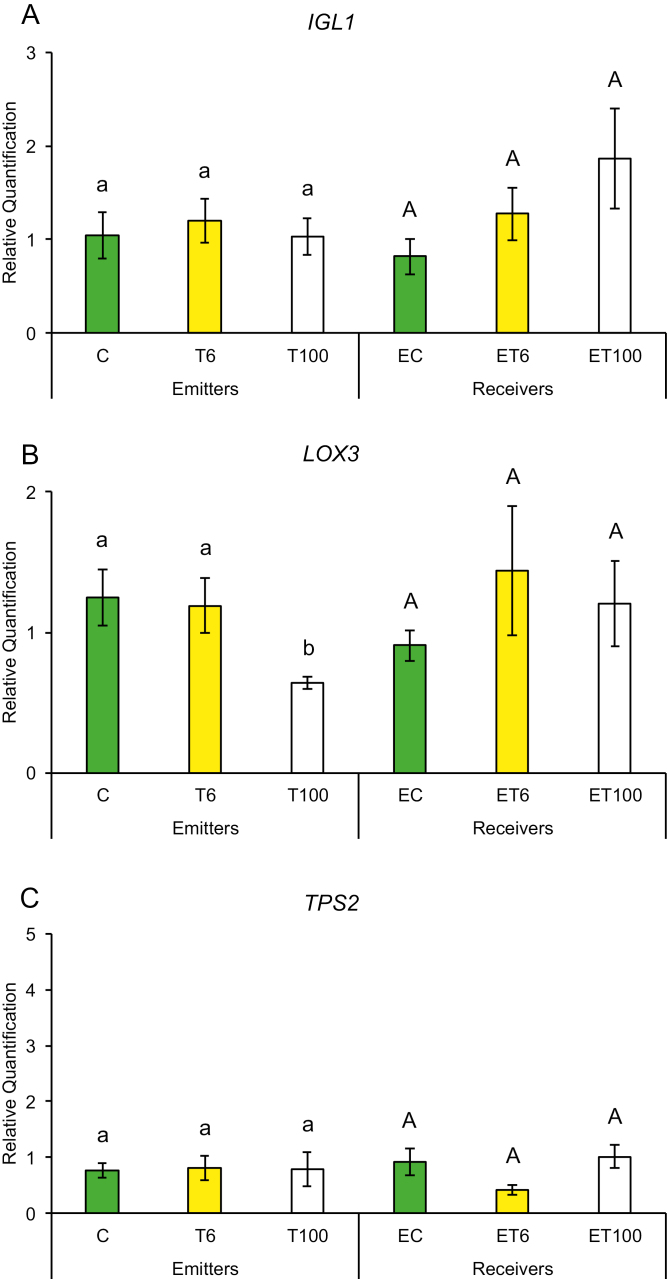
Expression of genes involved in the production of volatile compounds that prime neighboring plants after first treatment in untouched control plants (C), touched plants after 6 min (T6) and 100 min (T100), and neighbors exposed to volatiles released from untouched control plants (EC), touched plants after 6 min (ET6) and touched plants after 100 min (ET100). Different letters above each variable represent significant differences between treatments (Tukey’s test).

Promptly after first touch treatment, some early defense genes related to redox changes in the plant showed differences in transcript abundance compared with control treatments. At 6 min after touch treatment (T6), expression of the *SOD* gene was up-regulated and remained that way for 100 min after touch treatment (Fig 2D), while *CPK11* showed expression after 100 min exposure (ET100) (Fig 2B). Exposure to touch-induced volatiles for only 6 min was enough to up-regulate the *MPI* gene involved in direct chemical defenses against herbivores, which remained up-regulated after 100 min (Fig. 3B). The other genes, *BX1* and *WIP1*, responsible for the production of direct chemical defense against herbivores, did not show changes in expression in touched plants, nor in their exposed neighbors (Fig. 3A, C). Genes that are involved in priming, such as *IGL1* and *TPS2*, did not change in response to first touch (Fig. 4A, C). Interestingly, *LOX3*, which is involved in the synthesis of green leaf volatiles, showed suppression in its transcript abundance 100 min after touching (Fig. 4B).

**Fig. 5.**
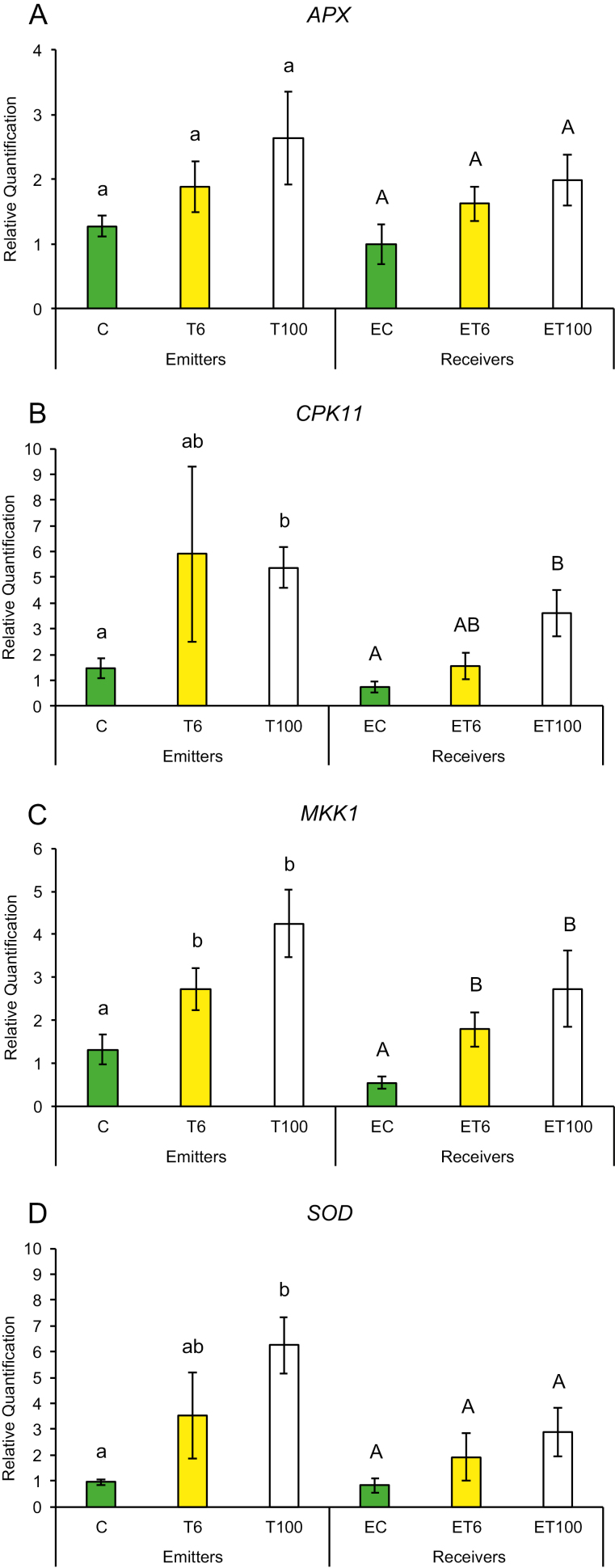
Transcript levels of early plant defenses related genes after six treatments in untouched control plants (C), touched plants after 6 min (T6) and 100 min (T100), and neighbors exposed to volatiles released from untouched control plants (EC), touched plants after 6 min (ET6) and touched plants after 100 min (ET100). Different letters above each variable represent significant differences between treatments (Tukey’s test).

**Fig. 6.**
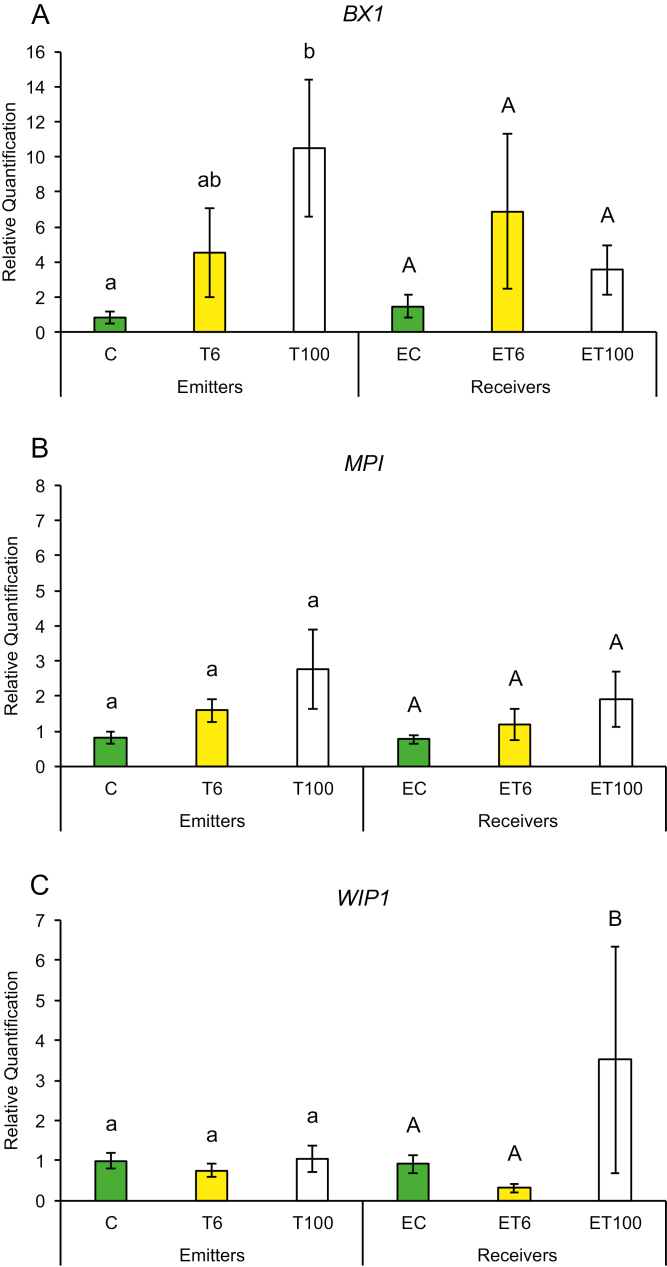
Changes in expression of genes involved in the production of chemical defense against herbivores after six treatments in untouched control plants (C), touched plants after 6 min (T6) and 100 min (T100), and neighbors exposed to volatiles released from untouched control plants (EC), touched plants after 6 min (ET6) and touched plants after 100 min (ET100). Different letters above each variable represent significant differences between treatments (Tukey’s test).

**Fig. 7.**
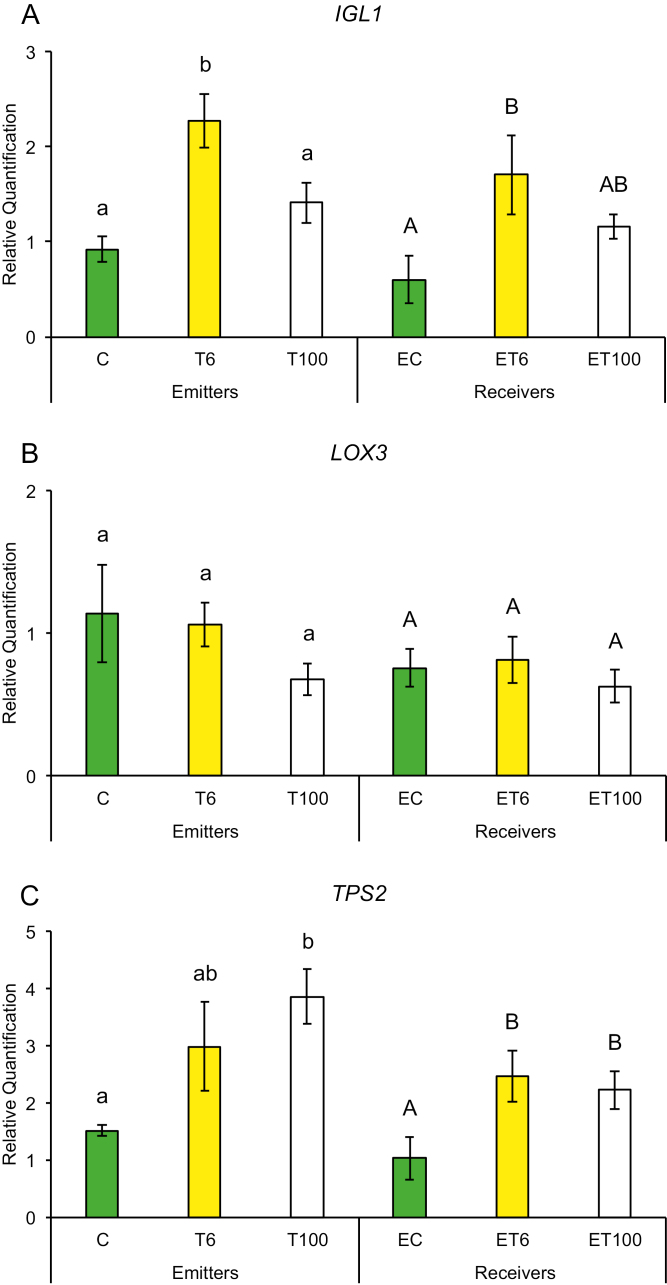
Expression profile of genes involved in the production of volatile compounds that prime neighboring plants after six treatments in untouched control plants (C), touched plants after 6 min (T6) and 100 min (T100), and neighbors exposed to volatiles released from untouched control plants (EC), touched plants after 6 min (ET6) and touched plants after 100 min (ET100). Different letters above each variable represent significant differences between treatments (Tukey’s test).

Plants that were touched for 1 min d^−1^ for 6 d showed significantly higher levels of the early activated defense-related gene *MKK1* at both time points T6 and T100 (Fig. 5C), while *CPK11* and *SOD* had higher transcripts at T100 compared with untouched plants (Fig. 5B, D). Direct chemical defense-related gene *BX1* (Fig. 6A) had higher transcripts 100 min after touch treatment, while genes involved in volatile synthesis such as *IGL1* and *TPS2* had higher transcript abundance 6 and 100 min after touching, respectively (Fig. 7A, C). Plants exposed to touch-induced volatiles for 6 min (ET6) after the last treatment (1 min d^−1^ for 6 d) showed higher transcripts of *MKK1*, *IGL1*, and *TPS2* (Figs 5C, 7A, C) compared with EC plants exposed to volatiles emitted by untouched controls, while *MKK1* and *TPS2* (Figs 5C, 7C) remained up-regulated 100 min after exposure. Two genes, *CPK11* and *WIP1*, showed higher transcripts only at the time point ET100 (Figs 5B, 6C). Exposure to induced volatiles from plants touched for the extended time (6 d) activated the same defense-related genes in the neighbors. We found transcriptional mirroring effects in expression of *IGL1* (Fig. 7A) at time point ET6, in *CPK11* (Fig. 5B) and *TPS2* (Fig. 7C) at ET100 and in *MKK1* at both time points ET6 and ET100 (Fig. 5C) in touched and exposed plants. Such an effect was also observed for the *APX* (Fig. 5A), *MPI* (Fig. 6B), and *LOX3* (Fig. 7B) genes, which did not show transcripts in either touched or exposed plants.

### Aphid settling on exposed maize plants

**Fig. 8.**
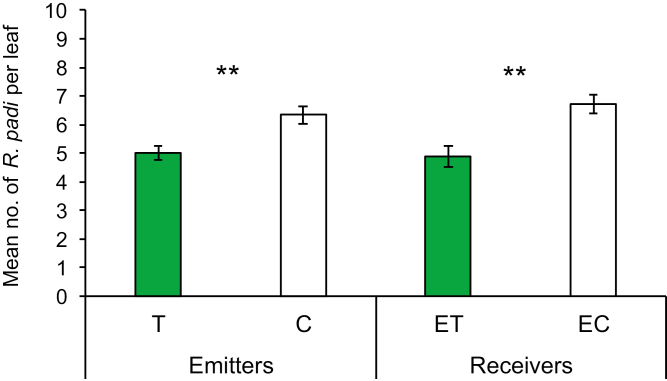
Aphid acceptance tests on emitter plants: touched (T) and untouched (C); and receivers exposed to volatiles released by touched maize (ET) and untouched maize (EC). Significant differences in aphid settling on tested plants are indicated (Tukey’s test, **P<0.01).

Aphid acceptance was significantly reduced in touched plants compared with untouched plants (*P*=0.001), and the same response was also observed for plant neighbors exposed to volatiles from touched plants in comparison with those exposed to volatiles from untouched plants (*P*=0.003) (Fig. 8). Results obtained in aphid settling tests demonstrated an important ecological role of contact-induced volatiles in chemical interactions among plants.

## Discussion

We found that, within minutes, plants can trigger transcription of defense-related genes in response to brief contact and consequently a rapid change in emission of volatile compounds. Surprisingly, the perception of induced VOCs from touched plants by neighboring plants can lead to the activation of defense genes. Taken together, our study demonstrates that genes activated in response to touch can contribute to induced resistance in the emitter itself and to primed/mirrored resistance in neighboring plants.

Plants touched for shorter periods of time as well as their neighbors showed a feeble defense response in gene expression at both time points (6 and 100 min). Besides searching for evidence in gene expression induced by touch, we aimed to identify active components in the volatile blend from touched plants that influenced up-regulation of defense-related genes in the volatile-exposed neighbors. After just 6 min of exposure, neighboring plants up-regulated direct defense-related gene *MPI* (Fig. 3B), and up-regulation of *CPK11* occurred after 100 min (Fig. 2B). The blend of touch-induced VOCs consisted of isoprene fragments, s4 aldehyde fragments, (*E*)-3-hexenal, and terpene fragments, which could be responsible for the observed receiver response (Fig. 1A). This demonstrates a fascinating ability of plants to rapidly detect, process, and respond to touch-induced changes in the volatile profile of their nearby neighbors. Further studies are needed to confirm the role of these VOCs in plant–plant communication as single components or together as a blend. However, sustained touching for 1 min d^−1^ for 6 d had a significant effect on gene expression in touched plants, and exposure to their induced VOCs, a blend of methanol, isoprene fragments, isoprene, alkyl fragments, and terpene fragments (Fig. 1B), activated the same genes in their neighbors.

Notably, the trend of such gene expression was mirrored between touched plants and their neighbors. For example, after 6 d of touching, *CPK11*, *IGL1*, *MKK1*, and *TPS2* transcripts were higher in both touched plants and plants receiving VOCs from these plants, compared with untouched plants or plants receiving VOCs from untouched plants, respectively (Figs 5B, C, 7A, C). This signifies two things. First, plants have a threshold at which they keep their induction of defense in response to touching to lower levels after 1 min of touching compared with sustained touching for 1 min d^−1^ for 6 d. Such a trend can be seen in the expression pattern of *LOX3* where there was a suppression of the transcript at 100 min compared with untouched plants on day 1, suggesting the presence of a possible checkpoint of one-time touch before activation of downstream volatile production to prime its neighbor to the imminent threat (Fig. 4B). Second, gene expression between touched plants and plants that receive VOCs from these touched plants can mirror their level of defense-related gene expression. This strongly suggests that the perception of specific VOCs emitted from touched plants can rapidly elevate defense levels in neighbors to mimic/copy the potentially adaptive transcripts of touched plants. This transcriptional mirroring response between touched plants and their neighbors for specific genes indicates that induced VOCs from perturbed plants can serve as messages to close conspecific neighbors. These results show plants’ capacity to sense and discriminate rapidly with great accuracy changes in nearby plants’ volatile profiles induced by touch, and to copy the response of plants directly exposed to mechano-stress. It is interesting to notice that this transcriptional mirroring effect is observable for certain genes of the plant early defense (*CPK11* and *MKK1*) (Fig. 5B, C) and of the volatile production (*IGL1* and *TPS2*) (Fig. 7A, C), but not for the selected genes involved in direct chemical defense against herbivores (Fig. 6A, C).

Plants are known to constantly monitor chemical signals as these signals often serve as an important source of information (Izaguirre *et al.*, 2006; Novoplansky, 2009; Kegge & Pierik, 2010). Plants benefit only if they are able to detect and respond in an appropriate manner to info-chemical signals from their environment (Broz *et al.*, 2010; Ninkovic, 2010). Receivers in close proximity utilize volatile cues leading to interactions between plants (Baldwin *et al.*, 2006; Ninkovic *et al.*, 2016).

It is apparent that 6 d of direct exposure significantly impacts the receivers’ ability to discriminately respond only to touch-induced volatiles. Such a response indicates that plants have the capacity to respond appropriately to the cues pointing to specific stress changes in their neighbors. This allows the eavesdropping plant to prepare in a timely fashion for future events that may have a consequence for plant performance, as observed in the similar response of aphids to directly bothered plants and to those that received this information. The production of VOCs is complex and constantly altered by interactions of plants with biotic factors (Pichersky *et al.*, 2006). VOCs present an indicator of the plant’s current state and may include elicitors of defense responses in neighboring plants (Karban *et al.*, 2014). Previous studies reported that genetic relatedness among sagebrush plants influences volatile herbivory-induced defense in receiving plants (Heil & Ton, 2008; Karban & Shiojiri, 2009; Das *et al.*, 2013; Sugimoto *et al.*, 2013; Erb *et al.*, 2015). Exposure to volatiles from insect-damaged conspecific plants can induce expression of several defense-related genes in undamaged neighbors (Arimura *et al.*, 2000). Our study demonstrates, for the first time, evidence that touch-induced volatiles from genetically identical plants can also play an important role in the induction of defense-related genes against herbivores in nearby neighbors.

Changes in volatile emission among the first and last touching treatments indicate that methanol, isoprene, and alkyl fragments might correlate with higher transcripts of *CPK11*, *IGL1*, *MKK1*, *TPS2*, and *WIP1* within exposed plants (Figs 5B, C, 6C, 7A, C). The expression of genes *CPK11*, *IGL1*, *WIP1*, and *MKK1* was associated to induced plant defense responses and greater resistance to insects and pathogens (Mészáros *et al.*, 2006; Kuśnierczyk *et al.*, 2008; Ahmad *et al.*, 2011; Szczegielniak *et al.*, 2012; Ding *et al.*, 2013; Gravino *et al.*, 2015). The expression of *TPS2* was significantly induced by corn leaf aphid feeding (Tzin *et al.*, 2015), enabling the production of monoterpenes and sesquiterpenes, including linalool and (*E*)-nerolidol (Richter *et al.*, 2016). Recent studies showed that sesquiterpenes such as (*E*)-nerolidol can increase emissions of plants exposed to volatiles from neighboring undamaged plants, which have strong repelling effects on the aphids (Ninkovic *et al.*, 2013; Dahlin *et al.*, 2015). The complex series of coordinated defense responses expressed in plants exposed to touch-induced volatiles reduced aphid settling, making these plants a less suitable host for aphids (Fig. 8). It is obvious that up-regulation of certain tested genes, usually related to plant-induced defense, can be also activated after exposure to volatiles from competing neighbors, indicating that these genes may have a physiological role in plant adaptation to growing environments. The ecological importance of touch-induced volatiles in induction of plants defenses is reflected in the fact that induction takes place even before actual herbivore attack occurs, making the exposed plants ready for a quick and more aggressive response.

We propose that brief and light contact events can be effective stimuli in the activation of transcripts of several defense-related genes that are also involved in the emission of specific volatiles that activate the same genes in exposed plants. This study presents evidence of induced volatiles as long-distance cues that play important roles in the chemical interactions among plants of the same genotype. The presented findings should be considered in further studies as potentially important factors and valuable mechanisms in plant–plant communication and in plant community interactions. Furthermore, the ecological costs, benefits, and constraints of such signaling are in need of further evaluation in this and additional contexts to fully understand if such signaling is adaptive for either the receiver or the emitter.

## Supplementary data

Supplementary data are available at *JXB* online.

Fig. S1. Exposure of maize plants to volatiles emitted from touched or untouched plants.

Table S1. List of primers used in this research.

## Supplementary Material

Supplementary Material
