# Supplementary material for: Airborne signals synchronize the defenses of neighboring plants in response to touch
Source: J Exp Bot. 2018 Oct 31;70(2):691–700. doi: 10.1093/jxb/ery375 (PMC6322579; doi:10.1093/jxb/ery375)
Supplement: Supplementary Material [file ery375_suppl_supplementary_material.pdf]

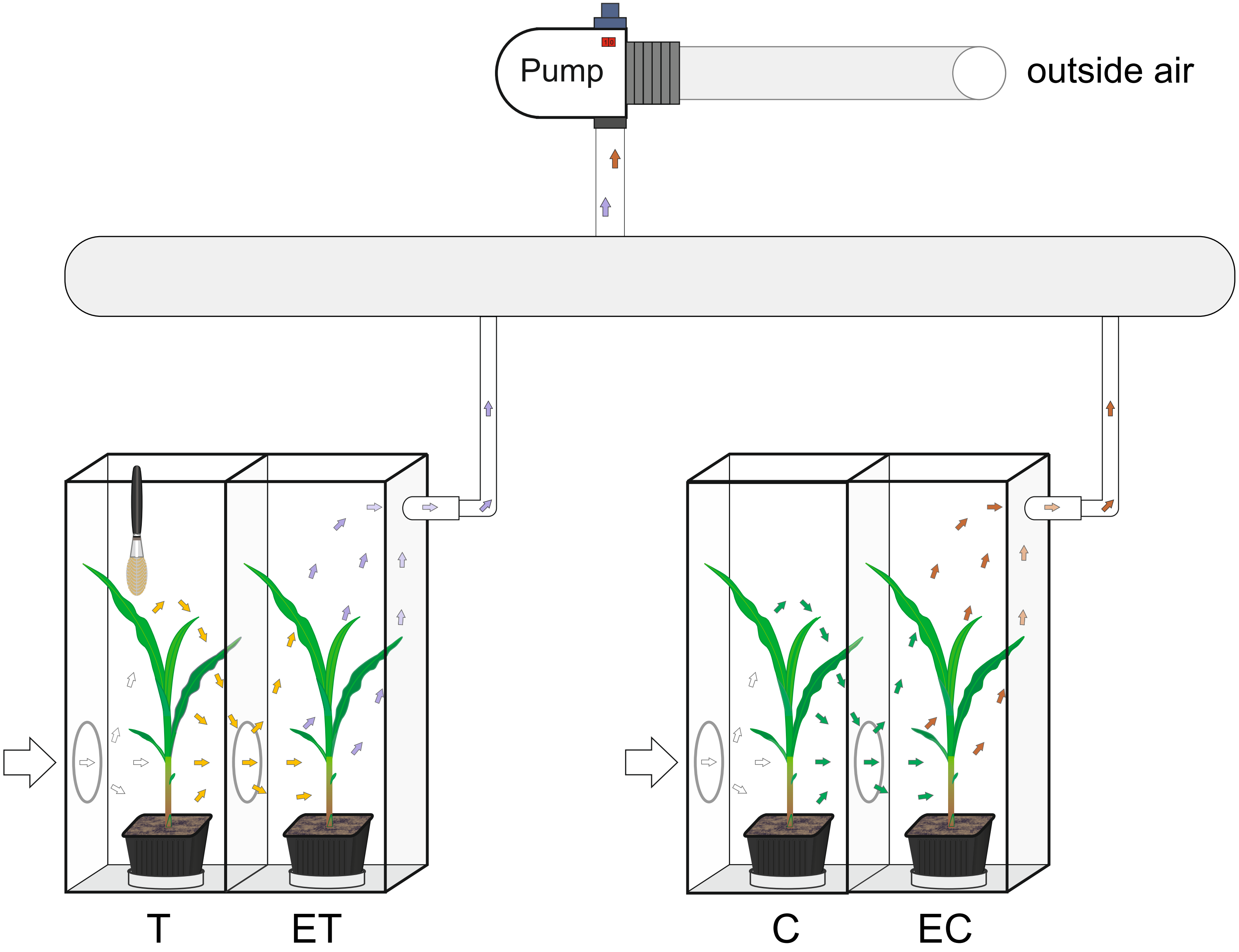


Fig. S1. Exposure of maize plants to volatiles emitted from touched or untouched plants.

Table S1. List of primers used in this research

| Gene name | Forward | Reverse | Maize GDB Accession/NCBI accession |
|---|---|---|---|
| APX | AGGCTGCTAGAGCCCATCAA | ACTGCCACGACTCCAGCAA | GRMZM2G140667 |
| CPK11 | TCCGTTTCTCGCTTCCATTC | AAAAGCCGACGAACGAAGCT | GRMZM2G047486 |
| MKK1 | GCCAATTATCTCGCCCTTTCA | TTTTGAGGCTCCCCTTTCCT | GRMZM2G400470 |
| SOD | GGTGTCAAGGGCACCATCTT | GGCTTGAGGCCAGAGACACT | GRMZM2G169890 |
| BX1 | CTCGACGAAGGAGGAACGAA | GGCGCGAAAGCCATGTC | GRMZM2G085381 |
| MPI (Chuang *et al.*, 2014) | GCGGATTATCGCCCTAACC | CGTCTGGGCGACGATGTC | X78988 |
| WIP1 (Chuang *et al.*, 2014) | AGCTCAAGTGCTGCACCAACT | GACGTCGTCGCAGGTGTAGA | X71396 |
| IGL1 | CGCCTTGCTCTCTTCTGGTT | CCGTTTGGGATCATCTTGTGA | GRMZM2G046191 |
| LOX3 (Chuang *et al.*, 2014) | GCTACGTACGAGCTGGTACATGAA | GCCGCTCTCTTCCCGTTT | AF149803 |
| TPS2 | GGTACATGTGGTCCATGGCAGC | GACGACGTAAACAAGCGAGATTATCTTGGT | GRMZM2G060669 |
| Actin (Chuang *et al.*, 2014) | GGAGCTCGAGAATGCCAAGAGCAG | GACCTCAGGGCATCTGAACCTCTC | U60511.1 |
